# Supplementary material for: Does darkness increase the risk of certain types of crime? A registered report protocol
Source: PLoS One. 2024 Jan 19;19(1):e0291971. doi: 10.1371/journal.pone.0291971 (PMC10798435; doi:10.1371/journal.pone.0291971)
Supplement: S1 File — (DOCX) [file pone.0291971.s001.docx]

**Supporting information 1 - Preregistration for analysis of secondary data (based on template developed by Akker et al, 2021)**

**Part 1: Study information**

**1.1 Working title of the study:** Does darkness increase the risk of certain types of crime? A Registered Report Protocol

**1.2 Authors of this preregistration:**

Jim Uttley (University of Sheffield) - ORCID iD: 0000-0002-8080-3473

Rosie Canwell (South Yorkshire Police)

Jamie Smith (South Yorkshire Police)

Sarah Falconer (South Yorkshire Police)

Yichong Mao (University of Sheffield) - ORCID iD: 0000-0003-3064-4600

Steve A Fotios (University of Sheffield) - ORCID iD: 0000-0002-2410-7641

**1.3 Research questions:**

RQ1: Does darkness increase the risk of crime occurring, after accounting for time of day and seasonal factors?

RQ2: Does the risk of a crime occurring after dark relative to during daylight vary depending on the type of crime?

RQ3: Does the risk of a crime occurring after dark relative to during daylight vary between different areas?

**1.4 Hypotheses:**

1) The overall risk of crime in South Yorkshire occurring after dark is greater than during daylight, after time of day and seasonal factors have been accounted for

2) The risk of a crime occurring after dark relative to during daylight will vary depending on the type of crime

3) The risk of a crime occurring after dark relative to during daylight is not uniform across Middle Super Output Areas within South Yorkshire

**Part 2: Data description**

**2.1 Datasets used**

This analysis uses crimes recorded by South Yorkshire Police, whose jurisdiction covers the Local Authority areas of Barnsley, Sheffield, Rotherham and Doncaster in the United Kingdom.

Crimes recorded as taking place between 1st January 2010 and 31st December 2019 will be included in the analysis. This time period covers the operation of two crime recording systems in South Yorkshire Police, CMS (Jan 2010 – Nov 2017) and CONNECT (Dec 2017 – Dec 2019). In both cases data is extractable via the SQL based system of Oracle BI. The data contains information about the crime category recorded. This categorisation is based on a hierarchy of offences laid down in the HOCR and is detailed by HMIC in their crime tree diagram (see S2 Fig). The study has opted for level 3 which strikes a balance of providing more detail to the nature of the crime versus the statistical power afforded/lost by aggregation/disaggregation. However, categories of crime that are classed as ‘other crimes against society’ (also known as ‘Crimes against the state’) will be excluded from the data. These crimes can often be the result of police generated activity i.e. the result of recording crime that wouldn’t have previously been reported because of proactive patrols/targeted interventions. The example that is most referred to being drug possession offences. These are often the result of a search or a targeted patrol and as such the more that activity occurs, the more the police record, meaning the indicator is a measure of police activity and not a base level of ‘true’ criminality.

Each crime record includes information about the time and date the crime was committed. A ‘Committed From Date’ and ‘Start time’ are recorded. Our analysis script combines these to create a ‘committed from’ time and date variable. A ‘Committed To Date’ and ‘End time’ are also recorded, and these are combined to create a ‘committed to’ time and date variable. For crimes when the exact time of committal are known, these two time and date variables are identical. Some crime records do not include a value for ‘End time’. In these instances it has been assumed the end time is identical to the start time. For crimes when the exact time of committal is not known, these provide the window of time the crime was potentially committed in.

The dataset of crime records will be filtered to only include crimes that were potentially committed during specific pairs of hours: 17:30-18:29 and 13:00-13:59; 18:30-18:29 and 14:00-14:59; 19:30-20:29 and 15:00-15:59. The later of the hours in each pair is labelled the ‘case’ hour - it will be in darkness for part of the year and in daylight for another part of the year. The earlier of the hours in each pair is labelled the ‘control’ hour - it is in daylight throughout the whole of the year. To filter the data in this way two inclusion criteria will be applied to crimes where the exact committal time was unknown, to determine their inclusion in the final dataset:

1) The midpoint between the ‘committed from’ and ‘committed to’ times should fall within the case or control hour

2) The difference between the ‘committed from’ and ‘committed to’ times should be less than one hour

Crime records that meet these two inclusion criteria will be included in the final dataset for analysis. Crimes where the exact committal time is known (i.e. the ‘committed from’ and ‘committed to’ times were the same) will also be included in the final dataset if this time falls within the case or control hour.

The crime data also includes the Middle Super Output Area (MSOA) where each crime took place. MSOAs are a layer of geographical areas in the UK designed to support the reporting of small-area statistics. MOSAs have a minimum population of 5,000, with a mean population of 7,200. As the definition of an MSOA boundary is based around population level they can vary greatly in area - being relatively small in densely populated areas but large in sparsely populated areas. The analysis will provide overall crime odds ratios for each MSOA in South Yorkshire. Odds ratios by crime type for each MSOA will not be calculated because this level of disaggregation is likely to lead to very small crime counts included in the odds ratio calculation, and potentially also produces ethical issues in terms of anonymity, with small count data potentially allowing the identification of victims.

A fictional example of unfiltered and unprocessed data that will be used in this analysis is shown in Table S1.

**Table S1.** Example of unfiltered, unprocessed fictional data.

| **Incident Number** | **HMIC Crime Tree Level 3** | **MSOA** | **Committed From Date** | **Committed To Date** | **Start time** | **End time** |
| --- | --- | --- | --- | --- | --- | --- |
| 3 | ALL OTHER THEFT OFFENCES | Hackenthorpe | 22/09/2018 | 24/09/2018 | 17:12 | 03:41 |
| 4 | THEFT FROM THE PERSON | Handsworth South | 20/01/2018 | 22/02/2018 | 02:05 | 02:05 |
| 5 | BURGLARY - DWELLING | Lower Stannington | 23/03/2018 | 23/03/2018 | 18:12 | 21:53 |
| 6 | BICYCLE THEFT | Crabtree & Fir Vale | 31/10/2018 | 31/10/2018 | 16:56 | 19:00 |
| 7 | ROBBERY - BUSINESS | Southy Green West | 11/05/2018 | 11/05/2018 | 19:59 | 21:23 |
| 8 | ARSON | Sharrow | 14/02/2018 | 14/02/202018 | 18:22 | Not Recorded |

**2.2 Data availability**

The dataset is not publicly available

**2.3 Data access**

The dataset can only be accessed by employees of South Yorkshire Police. A data extraction and analysis script was developed in R by researchers at the University of Sheffield (Uttley and Fotios) using a fictional example of data provided by South Yorkshire Police. This script will be passed to officers in South Yorkshire Police (Canwell, Smith and Falconer) who will apply it to their database of crime records. The R script produces an aggregated set of crime counts that will then be passed back to the researchers at the University of Sheffield for analysis and reporting. This process ensures data confidentiality is not breached. It also ensures access to the raw data by the University of Sheffield researchers who developed the hypotheses and analytical plan is restricted, ensuring prior knowledge of the data is limited.

**2.4 Data identifiers**

Not applicable - no URL, DOI or other persistent, unique identifier of the dataset is available as the data is not publicly available and is held confidentially by South Yorkshire Police.

**2.5 Access date**

Data has not been accessed yet.

**2.6 Data collection procedures**

The police record a crime through a variety of channels, most frequently through attending a report of an incident. The crime will be submitted for recording either by the operator dealing with the incident or the officer attending the scene. The crime will then be recorded by the Force Crime Bureau (FCB), a sub division of the control room, and then allocated to an officer to investigate further. The crime is recorded on the force crime system, CONNECT (formerly CMS), according to the crime recording processes outlined in the ‘Home Office counting rules for recorded crime’ (HOCR). More detail can be found here: <https://www.justiceinspectorates.gov.uk/hmicfrs/our-work/article/crime-data-integrity/crime-recording-process/>

South Yorkshire Police, as do all other Home Office affiliated forces, adhere to the HOCR and the National Crime Recording Standards (NCRS). This is to ensure crimes are recorded and counted in a standardised manner to allow for, amongst other reasons, a comparative overview of crime rates at a national and subnational level. In recording crime, there is oversight as to the accuracy of this process conducted within South Yorkshire Police via external and internal audit provided in part by inspections conducted by His Majesty’s Inspectorate of Constabulary (HMIC).

**2.7 Codebook**

Not applicable - no codebook is available for this dataset.

**Part 3: Variables**

**3.1 Manipulated variables**

Not applicable.

**3.2 Measured variables**

Not applicable - the dataset is observational data representing counts of crimes in different crime categories and in different geographical areas.

**3.3 Unit of analysis**

Counts of individual crime records will be aggregated into four distinct periods based on the hour and time of year they occurred (see Part 5 for a description of these periods). These four counts will be provided for each crime category. Crime counts in the four periods will also be provided for each MSOA (sub-district geographical area that can be considered as representing a ‘neighbourhood’) but these will be aggregated across all crime categories to avoid problems with small samples and the potential for violating data anonymity principles.

**3.4 Missing data**

Crime records will not be included in the analysis if they are likely to have been committed outside the time periods that are of interest (see section 2.1), or if no information is given about the time they were committed.

Crimes categorised as ‘Other crimes against society’ will not be included in the analysis. This includes drug offences, possession of weapons offences, public order offences and miscellaneous crimes against society (see S2 Fig).

**3.5 Statistical outliers**

We do not anticipate any statistical outliers within the data as it represents observational data based on official crime records.

**3.6 Sampling weights**

Not applicable.

**Part 4: Knowledge of data**

**4.1 Prior publication / dissemination**

The authors have not previously worked on any publications, papers or reports based on the specific data that will be extracted from crime recording systems of South Yorkshire Police.

**4.2 Prior knowledge**

Three of the authors (Smith, Canwell and Falconer) have access to and knowledge of the crime records database from which the data for this analysis will be extracted, as they are employees of South Yorkshire Police. However they do not have any prior knowledge of the exact set of data that will be extracted or about the relationship between light conditions and crime risk that the extracted data will be used to test.

Three of the authors (Uttley, Mao and Fotios) have no access to and no prior knowledge of the database from which the data in this analysis will be extracted, or the specific set of data that will be extracted.

**Part 5: Analyses**

**5.1 Statistical models**

This analysis will use odds ratios to assess the influence darkness has on the risk of crime. The dataset will be filtered to only include those that occur in pairs of case and control hours (see section 2.1). Counts of crimes will be aggregated into four groups, based on when they occurred:

CaseDark: Crimes that occurred during one of the case hours (17:30-18:29, 18:30-19:29 and 19:30-20:29) when that case hour was in darkness.

CaseDay: Crimes that occurred during one of the case hours when that case hour was in daylight.

ControlDark: Crimes that occurred during one of the control hours (13:00-13:59, 14:00-14:59 and 15:00-15:59) when that hour’s paired case hour was in darkness. For example, crimes that occurred during 13:00-13:59 would be included in this group if they occurred on a date when the paired case hour, 17:30-18:29, was in darkness.

ControlDay: Crimes that occurred during one of the control hours when that hour’s paired case hour was in daylight.

Whether a case hour is in daylight or darkness will be defined by day of the year, as set out in Table S2. On days of the year that fall outside the ranges for darkness and daylight shown in Table S2 the case hour will partially be in twilight. Any crimes that occur in case or control hours on such dates will be excluded from the analysis.

We define darkness as being when the sun’s altitude is at or below -6°. We choose this definition because this represents the transition between civil twilight (when the sun’s altitude is between -6° and 0°) and nautical twilight (when the sun’s altitude is between -6° and -12°). Based on data from solar monitoring sites in the UK, as reported in Raynham et al [28], the average illuminance when the sun’s altitude is at -6° is only 2.33 lx.

We define daylight as being when the sun’s altitude is at or above 0°. This altitude represents the time of sunrise or sunset. Based on the solar illuminance data reported in Raynham et al [28], the average illuminance at this altitude is 509 lx.

The period when the sun’s altitude is between -6° and 0° is defined as twilight and represents a transition between ambient daylight and ambient darkness.

**Table S2.** Days of the year that define whether case hour is in daylight or darkness.

| **Case hour** | **Days of year case hour is in darkness** | **Days of year case hour is in daylight** |
| --- | --- | --- |
| 17:30-18:29 | 1-33, 304-365 (or 366 if leap year) | 87-278 |
| 18:30-19:29 | 1-65, 296-365 (or 366 if leap year) | 90-253 |
| 19:30-20:29 | 1-83, 270-365 (or 366 if leap year) | 120-227 |

An odds ratio will be calculated for each crime category. This odds ratio will represent the odds of a crime being committed after dark compared with during daylight. The odds ratio will be calculated using the CaseDark, CaseDay, ControlDark and ControlDay counts, as shown in equation 1. A confidence interval for each odds ratio will be calculated using equation 2.

*OddsRatio =* $\frac{CaseDark}{CaseDay} \div\frac{ControlDark}{ControlDay}$ (1)

*95% CI =* $exp\left( ln\left( OddsRatio \right)\pm1.96\sqrt{\frac{1}{CaseDark}+\frac{1}{CaseDay}+\frac{1}{ControlDark}+\frac{1}{ControlDay}} \right)$ (2)

Where:

*CaseDark* = Count of crimes in case hour when it is in darkness

*CaseDay* = Count of crimes in case hour when it is in daylight

*ControlDark* = Count of crimes in control hour when case hour is in darkness

*ControlDay* = Count of crimes in control hour when case hour is in daylight

Odds ratios will also be calculated for each Middle Super Output Area. These odds ratios will be calculated from counts of crimes aggregated across all crime categories, to avoid counts that are too small for the calculation of meaningful odds ratios, and to avoid the potential identification of victims involved in crimes.

We will test our first hypothesis, that the overall risk of crime in South Yorkshire occurring after dark is greater than during daylight, by calculating a single odds ratio that incorporates counts in all categories of crimes combined. The hypothesis will be confirmed if the odds ratio is statistically significantly greater than 1.0.

We will test our second hypothesis, that the risk of a crime occurring after dark relative to during daylight is not uniform across Middle Super Output Areas, by calculating odds ratios for each MSOA, using counts of crimes aggregated across all crime categories for each MSOA. The hypothesis will be confirmed if any of the 95% confidence intervals for each MSOA odds ratio do not overlap.

We will test our third hypothesis, that the risk of a crime occurring after dark relative to during daylight will vary depending on the type of crime, by calculating separate odds ratios for each category of crime. The hypothesis will be confirmed if any of the 95% confidence intervals for each crime category odds ratio do not overlap.

**5.2 Effect size**

Based on analyses of the effect of darkness on crime counts in previous work [19, 20] we anticipate odds ratios between 0.5 and 2.5. For example, Fotios et al [19] found an overall odds ratio for all crime of 1.05 (95% confidence interval: 1.01-1.10), and odds ratios for different crimes ranging between 0.67 and 2.04, although only the odds ratios for robbery was found to be statistically significantly different to 1.0.

**5.3 Statistical power**

The epi.ssc function from the *R* package *epiR* was used to estimate the total count of crimes in the case and control periods required to detect odds ratios of sizes ranging between 1.2 and 2.5, with a minimum power of 80%, a confidence level of 95%, and a one-sided test. These are shown in Table S3. In calculating these required counts the following assumptions were made, based on the data reported in Fotios et al [19]:

1) The proportion of crimes in the control hour when it was in darkness was 50% of all crimes recorded in the control hour

2) The total count of crimes in the control hour was 2.3 times that of the total count of crimes in the case hour

**Table S3.** Required total counts in case and control hours to detect different odds ratios with 80% power.

| **Odds ratio** | **Count required in case hour** | **Count required in control hour** |
| --- | --- | --- |
| 1.2 | 1070 | 2461 |
| 1.5 | 219 | 504 |
| 1.8 | 105 | 242 |
| 2.1 | 67 | 155 |
| 2.4 | 49 | 113 |

**5.4 Inference criteria**

To confirm hypothesis 1 we will calculate a p-value associated with the odds ratio associated with all crime, and use a threshold of 0.05 to indicate statistical significance.

To confirm hypotheses 2 and 3 we will calculate 95% confidence intervals associated with the odds ratios for all MSOAs (hypothesis 2) and for all crime categories (hypothesis 3), using equation 2. Non-overlapping confidence intervals will be used to indicate differences between odds ratios, either between different MSOAs or between different crime categories.

**5.5 Assumption violation / model non-convergence**

Odds ratios cannot be calculated if any of the four required counts are zero. Therefore, if one of the four counts is zero, 0.5 will be added to all four counts used in the odds ratio, following the Haldane-Anscombe correction that is commonly used in such cases for odds ratios (e.g. see [30]). If more than one of the four counts is zero, the odds ratio will not be calculated.

**5.6 Reliability and robustness testing**

The results may be sensitive to the choice of case and control hour. We will therefore carry out a sensitivity analysis using a combination of two case hours in the morning, 05:00-05:59 and 06:00-06:59, paired with control hours of 11:00-11:59 and 12:00-12:59 respectively. The days of the year that these two case hours will be in daylight or darkness are shown in Table S4. This sensitivity analysis will help show whether the odds ratios reported in the main analysis may be sensitive to the choice of case and control hours or not.

**Table S4.** Days of the year that define whether sensitivity analysis case hours are in daylight or darkness.

| **Case hour** | **Days of year case hour is in darkness** | **Days of year case hour is in daylight** |
| --- | --- | --- |
| 05:00-05:59 | 1-67, 90-91, 257-365/366 | 141-196 |
| 06:00-06:59 | 1-39, 291-297, 325-365/366 | 84, 110-234 |

**5.7 Exploratory analysis**

No additional exploratory analysis is planned.
