# Supplementary material for: Does darkness increase the risk of certain types of crime? A registered report protocol
Source: PLoS One. 2024 Jan 19;19(1):e0291971. doi: 10.1371/journal.pone.0291971 (PMC10798435; doi:10.1371/journal.pone.0291971)
Supplement: Attachment — Submitted filename: Response to reviewers - second submission, FINAL.docx [file pone.0291971.s005.docx]

**“Does darkness increase the risk of certain types of crime? A Registered Report Protocol” – Response to reviewers**

| **Reviewer 1** | | |
| --- | --- | --- |
| **Comment ID** | **Comment** | **Response** |
| 1.1 | I do not think the methodology is sufficiently rigorous because looking at daytime, evening, and nighttime comparisons misses the important attributes of crime, including the time of offence literature. For example, we know that crime concentrates in the afternoon hours because juveniles come out of school and interact with other juveniles. But the ones that commit crimes at night are different. We also know that there are different structural opportunities to commit crime at different hours of the day, depending on, for example, the availability of victims. We have also learned that there are hotspots of crime that are affected by ecological factors and are conditional on the time of day (i.e., shopping centres or cars to break into). In short, the methodology will miss all that. | We agree with the reviewer that time of day is an important factor in terms of the type of crime being committed. We have added text and citations that describes the variation in crime counts by time of day, for different crimes (see lines 166-178).  However, the reviewer appears to have misunderstood the aim of this method. We take instances of crime and ask whether that same crime is likely to have taken place had the ambient light level at that same time changed from darkness to daylight or vice versa. Rather than ignoring the influence of time of day on crime, we controlled time of day and examined only those crimes within a set period of each day.  We have also added text to section 2.5 in relation to the sensitivity analysis, as this analysis uses different case and control hours to those used in the main analysis. This will allow us to see whether time of day has an influence on how darkness affects crime. |
| 1.2 | There is not much that can be done by way of statistical controls on the same units. Instead, attempt to find areas that are as close as possible to one another where some have lighting and some do not, and if you can then control for confounding variables through randomization, even better. If randomization cannot be achieved, then (a) find hotspots of crime; (2) separate them by light/no light or different levels of light or perhaps different types of lights; and then try to do propensity score matching or some other technique to contrast the two groups. | We appreciate the reviewer’s suggestions. However, these relate to comparing locations with different (street) lighting conditions. Our study proposes to assess the effect of darkness compared with daylight on crime, not the effect of lighting after dark on crime. We agree that examining the effect of lighting on crime is worthwhile and should be carried out in future work, a first step towards this is establishing the effect darkness has on crime. If no effect is found, it is unlikely that any effect of lighting after dark would be found (assuming the key factors to be changes in visibility and perceived visibility). This justification of first focusing on the effect of darkness is explained on lines 146-161, with the text revised slightly for improved clarity (lines 159-161).  The case / control approach we propose to take does act as a statistical control. Using a specific case hour to compare daylight and darkness controls for the influence of time of day on crime. Using a control hour controls for the influence of the time of year and seasonal effects such as weather conditions on crime. One part of our analyses will calculate odds ratios for all MSOA areas in the region. The MSOA therefore acts as its own control as we compare counts of crimes in the same area but during daylight and darkness. Text has been added to section 1 to make this point (see lines 260-263). |
| **Reviewer 2** | | |
| **Comment ID** | **Comment** | **Response** |
| 2.1 | I would like to see the outputs of the analysis of the fictional data, ideally in the main text. Ideally, with multiple sets of fictional data that would represent a range of support for/against the various hypotheses. | Tables of fictional data added, with a description of the conclusions that would be drawn from these results. We have also described what results would look like if alternative conclusions were to made about our three hypotheses. See lines 513-543. We have not included actual data for these alternative conclusions though as this would require additional large tables of data within the manuscript, and additional supplementary files with the alternative fictional data, for minimal additional explanatory benefit. |
| 2.2 | S1-5.3 - how does this compare with the total amount of data available? What range of odds ratios do you have power to detect?  Might want to bump/copy this section to main - it's important | Text from S1-5.3 has now been included in the main manuscript (section 2.6). Additional text added explaining our estimates of the total crime counts we might expect, and the size of odds ratio this would allow us to detect (see lines 577-593). |
| 2.3 | "data collected during the study will be made fully available without restriction upon study completion" - I think this requires clarification. If I've understood correctly, the raw data will not be made available. What will be? | Apologies for the confusion – we agreed with this statement in reference to the fictional data that has been used within the manuscript to demonstrate our analysis.  We are unable to make the real raw data openly available as the details within it (date and time of crime, crime category, and location) could potentially allow the identification of individuals involved in a crime.  We have added a data availability statement to the manuscript to clarify this (see lines 608-615). |
| 2.4 | "Level 3" needs to be defined in text. Perhaps bump S2 to main? | Text amended and crime tree diagram inserted into main text as Figure 1. See lines 412-418. |
| 2.5 | S1, eq2 is broken | This may have been caused by the manuscript compilation process of the publishers. We have uploaded the S1 document as a pdf to ensure the equation is visible. |
| 2.6 | "reduced significantly less" - confusing wording | Text amended (see lines 108-110). |
| 2.7 | I'm surprised there isn't any info considering the issue from the perspective of offenders in terms of how light effects motivation/ability to commit crime. Is there a literature on this? | Text added to Introduction, discussing some literature related to offender perceptions and decision-making, and 4 new references added. This literature is consistent with the literature already described about crime counts - that there is not a consistent relationship between light levels and crime committal. See lines 69-86. |
| 2.8 | “If the risk of crime occurring is shown to be higher after dark than in daylight in an area, effective lighting for crime reduction would reduce this relative increase in risk after dark.”  I think this statement needs to acknowledge remaining potential confounds as caveats | Text amended (see lines 159-178), and additional references added in relation to other contributory factors to crime that may act as confounds. To be clear, we are suggesting that a measure of the risk of crime being committed after dark compared with during daylight could be used to evaluate whether lighting for crime prevention is effective, by seeing whether this measurable risk reduces following the lighting intervention. We are not suggesting lighting will reduce the risk – this remains an empirical question that we do not directly address in this study. |
| 2.9 | It might be worth considering adding a contingency table pre-eq1, and using the standard nomclomenture in eq1 (OR = AD/BC). | We have added the suggested contingency table as Table 1 and amended relevant text, see lines 360-362 and 368-369. We would prefer to continue using the terms CaseDark, CaseDay etc. for clarity, as these terms are referred to later in the manuscript. |
| 2.10 | Example OR values and associated conclusions, for demonstration purposes? | We have now included example output, including OR values, and their interpretation (see response to comment 2.1, and lines 513-543). |
| 2.11 | "the case hours being" - just ref table 1 rather than repeat? | Text amended (see line 445). |
| 2.12 | Aoristic defined twice | Second definition removed (line 453). |
| 2.13 | Seems to assume that all crimes occur in less than 1 minute - isn't some of the difference between start and end time would be the length of time that the crime was taking place, rather than just uncertainty? | Text amended to acknowledge different start and end times could reflect the length of time a crime occurred for. It is not possible to distinguish such crimes from aoristic crimes however, so we therefore treat both the same way. See lines 454-460. |
| 2.14 | L451 might want to note that 0.05 implemented by 1.96 in eq2 | The 0.05 referred to on this line relates to the significance threshold for the p-value associated with the odds ratio. The 1.96 value in eq2 relates to the alpha used for calculation of the confidence intervals. We think these are two separate (but related) things. For added clarity, we have added text explaining we will be using Fisher’s exact test to calculate a p-value to assess hypothesis 1, see lines 501-502. |

In addition to the changes made to the manuscript in response to the reviewer comments, as described above, we have also made a small number of proofing edits and corrections:

- Line 27 – the word ‘potential’ added to the abstract
- Lines 97-98 – the words ‘the authors of that work included’ added to text
- Line 131 – the word ‘second’ added to text
- Lines 267-268 – the words ‘such as illuminating a previously unlit route,’ added to text
